# Impact of point-of-care testing on the management of sexually transmitted infections in South Africa: Evidence from the HVTN702 HIV vaccine trial

**DOI:** 10.1093/cid/ciac824

**Published:** 2022-10-15

**Authors:** Kwabena Asare, Tsion Andine, Nivashnee Naicker, Jienchi Dorward, Nishanta Singh, Elizabeth Spooner, Jessica Andriesen, Farzana Osman, Sinaye Ngcapu, Alain Vandormael, Adrian Mindel, Salim S. Abdool Karim, Linda-Gail Bekker, Glenda Gray, Lawrence Corey, Andrew Tomita, Nigel Garrett

**Affiliations:** 1 Discipline of Public Health Medicine, School of Nursing and Public Health, University of KwaZulu-Natal, Durban, South Africa; 2 Centre for the AIDS Programme of Research in South Africa (CAPRISA), University of KwaZulu-Natal, Durban, South Africa; 3 Health Economics and HIV and AIDS Research Division (HEARD), University of KwaZulu-Natal, Durban, South Africa; 4 Department of Internal Medicine, College of Medicine, Howard University, Washington, District of Columbia, USA; 5 Nuffield Department of Primary Care Health Sciences, University of Oxford, Oxfordshire, United Kingdom; 6 HIV and Other Infectious Diseases Research Unit, South African Medical Research Council, Cape Town, South Africa; 7 Vaccine and Infectious Disease Division, Fred Hutchinson Cancer Research Center, Seattle, US; 8 Department of Medical Microbiology, School of Laboratory Medicine and Medical Sciences, University of KwaZulu-Natal, Durban, South Africa; 9 Heidelberg Institute of Global Health, Heidelberg University, Heidelberg, Germany; Department of Medical Microbiology, School of Laboratory Medicine and Medical Sciences, University of KwaZulu-Natal, Durban, South Africa; 10 The Desmond Tutu HIV Centre, University of Cape Town, Cape Town, South Africa; 11 Centre for Rural Health, School of Nursing and Public Health, University of KwaZulu-Natal, Durban, South Africa; 12 KwaZulu-Natal Research Innovation and Sequencing Platform (KRISP), College of Health Sciences, University of KwaZulu-Natal, Durban, South Africa

**Keywords:** Sexually transmitted infections, Point-of-Care testing, Central laboratory-based testing, treatment initiation, Adverse event reporting

## Abstract

**Background:**

Alternative approaches to syndromic management are needed to reduce high rates of sexually transmitted infections (STI) in resource-limited settings. We aimed to determine the impact of point-of-care (POC) versus central laboratory-based testing on early STI treatment initiation and reporting of adverse events (AEs) that were STIs (STI-AE).

**Methods:**

We used Kaplan-Meier and Cox regression models to compare times to STI treatment initiation and STI-AE reporting among HVTN702 HIV vaccine trial participants at three research clinics in South Africa. *Neisseria gonorrhoeae* (NG) and *Chlamydia trachomatis* (CT) were diagnosed with POC assays at eThekwini clinic and with central laboratory-based systems at Verulam and Isipingo clinics. All clinics used POC assays for *Trichomonas vaginalis* (TV) testing.

**Results:**

Among 959 women (median age 23 years, IQR 21-26), median days (95%CI) to NG/CT treatment initiation and NG/CT-AE reporting were 0.20 (0.16-0.25) and 0.24 (0.19-0.27) at eThekwini versus 14.22 (14.12-15.09) and 15.12 (13.22-21.24) at Verulam/Isipingo clinics (all p<0.001). Median days (95%CI) to TV treatment initiation and TV-AE reporting were 0.17 (0.12-0.27) and 0.25 (0.20-0.99) at eThekwini versus 0.18 (0.15-0.2) and 0.24 (0.15-0.99) at Verulam/Isipingo clinics (all p>0.05). Cox regression analysis revealed that NG/CT treatment initiation (aHR=39.62, 95%CI 15.13-103.74) and NG/CT-AE reporting (aHR=3.38, 95%CI 2.23-5.13) occurred faster at eThekwini compared to Verulam/Isipingo clinics, while times to TV treatment initiation (aHR=0.93, 95%CI 0.59-1.48) and TV-AE reporting (aHR=1.38, 95%CI 0.86-2.21) were similar between clinics.

**Conclusions:**

POC testing led to prompt STI management with potential therapeutic and prevention benefits, highlighting the utility as diagnostic tools in endemic resource-limited settings.

## Introduction

Sexually transmitted infections (STIs) continue to affect large populations globally, despite the availability of effective treatment[1–4]. The World Health Organization (WHO) estimates that 374 million new infections of curable *Neisseria gonorrhoeae* (NG), *Chlamydia trachomatis* (CT), *Trichomonas vaginalis* (TV) and *Treponema pallidum* occur annually among adults aged 15-49 years worldwide, of which 63 million (16%) are recorded in the WHO-Africa region[5].

These curable STIs are associated with pelvic inflammatory disease, ectopic pregnancy, infertility, genital ulcerations, foetal and neonatal complications, including deaths[2, 4, 6–8], and HIV transmission risk[9].

Many years after the introduction of syndromic management for STI care[10], the population-level STI burden remains high in sub-Saharan Africa (SSA)[5]. Syndromic management entails the identification of STI syndromes (e.g., vaginal and urethral discharge syndromes) and providing treatment to deal with the commonly suspected pathogens[1]. This approach has a low implementation cost and allows promptness in STI treatment, leading to its recommendation for resource-limited settings by the WHO in the 1990s[1]. However, due to the asymptomatic state of many STIs[1, 11, 12], particularly among women[13], and the low diagnostic accuracy of syndromic management[12, 14–16], high rates of untreated STIs and overuse of antibiotics[13] have been reported in most SSA settings[12, 14].

Diagnostic STI testing is arguably the best approach to management[17], but the gold standard laboratory-based polymerase chain reaction (PCR) testing usually has substantial operational costs and is hence less sustainable for resource-limited settings[1]. Moreover, central laboratory-based assays can cause delays in STI treatment initiation[18] due to long turnaround times for result availability[17, 19, 20]. Thus, a diagnostic method that possesses the strengths of diagnostic accuracy, prompt result turnaround, early treatment initiation or management, and cheaper operational costs would be ideal for clinical STI care in resource-limited settings.

Recent developments in diagnostic technologies have enabled an influx of potentially cheaper and more sensitive point-of-care (POC) assays for clinical care[21–23]. In South Africa, POC assays such as the Xpert CT/NG (Cepheid, Sunnydale, California, USA) and OSOM TV (Sekisui Diagnostics, Lexington, Massachusetts, USA) have demonstrated high diagnostic sensitivity (NG=100%, CT=100%, TV=75.0%) and specificity (NG=100%, CT=97.6%, TV=100%) when compared to standard laboratory-based PCR assays[24]. Similar findings have been recorded with different POC assays globally[25, 26]. However, it is still unclear whether POC assays' real-time diagnosis strength practically translates into earlier STI management compared to central laboratory testing to inform policy in resource-limited settings. To our knowledge, no study in SSA has yet compared POC and laboratory-based diagnostic approaches to determine their impact on early STI treatment initiation or other management outcomes. Therefore, recent calls for healthcare systems in resource-limited settings to consider POC testing as an alternative diagnostic care solution [10–12, 23] lack evidence regarding the effectiveness of POC versus laboratory-based testing on prompt STI management.

The objective of this study was to determine the relative effects of POC versus central laboratory-based testing on times to STI treatment initiation and reporting of adverse events (AEs) that were STIs in a cohort of women and men followed prospectively for 3 years in a phase 2b/3 HIV vaccine trial in South Africa.

## Methods

### Study design, population and setting

The randomised, double-blind, placebo-controlled HVTN702 trial was conducted between 2017 and 2020 to assess the efficacy of an ALVAC and bivalent subtype C gp120 HIV vaccine regimen adjuvanted with MF59 in South Africa. Briefly, the trial enrolled 5400 HIV-negative adults aged 18 to 35 years. Of these, 2700 were assigned to vaccine and 2700 to placebo at 14 clinical research sites (CRSs). The HVTN702 vaccine regimen did not prevent HIV-1 acquisition[27].

This sub-analysis included HVTN702 vaccine and placebo participants enrolled at three research clinics in eThekwini, Isipingo and Verulam in the KwaZulu-Natal province. These three clinics are based in the eThekwini Metropolitan area, which ensured homogeneity of any unobserved structural factors that could confound the results. The eThekwini clinic is based in central Durban, while the Isipingo and Verulam clinics are located on the outskirts of Durban. STI testing and treatment were performed at enrollment and every six months afterwards until study exit. The eThekwini CRS used POC assays, and the Isipingo and Verulam CRSs used a central laboratory-based system for NG/CT testing, but all three CRSs used POC assays for TV diagnosis. The HVTN702 trial was approved by the appropriate regulatory bodies in South Africa[27], and the Biomedical Research Ethics Committee of the University of KwaZulu-Natal, South Africa, approved this sub-analysis (BREC/00003808/2022).

### Study measures and assessments: STI testing, treatment initiation and STI-AE reporting

Experienced trial staff at eThekwini CRS collected vaginal and cervical swabs or urine samples for CT and NG testing with the Xpert CT/NG POC assay operated on the GeneXpert system. At the Isipingo and Verulam CRSs, vaginal and cervical swabs or urine samples were transported on wet ice to a central laboratory at the South African Medical Research Council in Durban for NG/CT testing on the GeneXpert system. Specimen transport to the central laboratory was twice daily at mid-day and afternoon, and the results turnaround was within 72hrs from receipt of the sample. The OSOM TV assay was used for POC diagnosis of TV on collected vaginal and cervical swab samples among women. All STIs were appropriately treated based on the diagnostic test results.

HVTN702 trial participants were monitored for AEs, including AEs that were STIs. These were any AE with a confirmed diagnosis of NG, CT or TV using a validated assay. We termed these as STI-AE(s) throughout the manuscript. STI-AE reporting time was when the CRSs received the laboratory result. The CRSs reported all collected AEs to the trial's statistical and data management centre using case report forms (CRFs). All study events and timings for sample collection, treatment initiation and STI-AE reporting were recorded based on quality assurance guidelines as part of the trial.

### Statistical analyses

All data analyses were conducted with STATA statistical package version 17 (StataCorp LLC, Texas, USA)[28]. We compared eThekwini CRS to Verulam/Isipingo CRSs on the study outcomes in women and men. First, we summarised and compared the demographic profiles of enrolled participants using Chi-squared (for proportion) and rank-sum (for median) tests to ascertain baseline comparability. We then used Chi-squared tests to compare the study outcomes, including the percentage of STI cases and times to STI treatment initiation and STI-AE reporting. We evaluated the impact of POC versus central laboratory-based testing on prompt STI treatment initiation and STI-AE reporting with time-to-event models.

The analysis time for all time-to-event models started at the time of sample collection and ended with study event occurrence (STI treatment initiation or STI-AE reporting) or censoring (study exit or the next sample collection observation during follow-up). We used the Kaplan-Meier estimate of the hazard function to compare trends in the cumulative probabilities of NG/CT treatment initiation and NG/CT-AE reporting with log-rank tests. We used Cox proportional hazard regression to determine the relative effects of POC compared to laboratory-based testing on times to NG/CT treatment initiation and NG/CT-AE reporting. We repeated the Kaplan-Meier and Cox regression analyses to compare times to TV treatment initiation and TV-AE reporting to confirm the consistency of the impact of POC testing on STI management since all CRSs used POC assays for TV testing. We performed a sensitivity analysis of the Cox models disaggregating all CRSs to identify any confounding of results due to aggregating Verulam and Isipingo CRSs data.

The longitudinal design of HVTN702 yielded repeat STI testing observations per participant. We adjusted for repeated observations with robust estimation of standard errors and used Breslow’s method to account for tied survival times in all Cox regression models[29]. We evaluated the goodness-of-fit of the Cox regression models with proportional hazard tests using Schoenfeld's residuals and adjusted models with time-varying covariate specifications when required[30, 31]. We used a 5% level of significance in all hypothesis tests.

## Results

### Baseline profile of enrolled women

A total of 959 women were enrolled and tested for STIs (NG, CT and TV) over a median of 4 visits (IQR 3-5) between March 2017 and June 2020 (median follow-up 2.3 years, IQR 1.7-2.9). The baseline demographic details and STI prevalence stratified by CRS are presented in Table 1. The median age was 23 years (IQR 21-26), and 60.3% were <25 years old. Most women (96.4%) indicated being married or having a stable sexual partner. At enrolment into the trial, the prevalence of NG, CT and TV were 3.3%, 19.8%, and 4.7%, respectively. Baseline characteristics of women across clinics were similar according to age categories, educational status, race/ethnicity and STI (NG, CT and TV) prevalence (all p>0.05).

### Overall STI testing, incidence, prevalence, treatment initiation and STI-AE reporting among women

The total number of STI tests performed during the trial, incidence, the percentage of positive cases, the corresponding percentage of treatment initiation and STI-AE reporting are shown in Table 2. The overall percentage of NG, CT, and TV cases during follow-up were 3.7%, 13.5% and 3.1%, respectively. Of those diagnosed with NG, CT, and TV, 76.9%, 94.2%, and 89.7% received appropriate treatment, respectively. Some participants, who were unable to wait for results, did not receive treatment in the trial because they never returned for follow-up. Furthermore, 72.0%, 65.4%, and 58.1% of NG, CT, and TV diagnoses were reported as AEs. STI incidence, percentage of cases, the percentage of treatment initiations, and STI-AEs reported were similar across CRSs (all p>0.05).

NG/CT treatment initiation and NG/CT-AE reporting were faster at eThekwini than Verulam/Isipingo CRSs (all p<0.05). Most NG/CT cases (92.4%) at eThekwini CRS received appropriate treatment on the day of testing compared to 1.2% at Verulam/Isipingo CRSs (p<0.001). The 7.6% of NG/CT positive participants at the eThekwini clinic who did not initiate treatment on the day of testing were unable to wait for results but returned for treatment at a future study visit. At Verulam/Isipingo CRSs, the non-same-day NG/CT treatment initiations occurred within 2-7 days (7.0%), 8-14 days (36.6%), and after 14 days (55.2%) of sample collection.

Furthermore, 98.5% NG/CT-AEs were reported on the day of testing at eThekwini compared to 4.8% at Verulam/Isipingo CRSs (p<0.001). At Verulam/Isipingo CRSs, the non-same-day NG/CT-AEs were reported within 2-7 days (30.9%), 8-14 days (37.1%), and after 14 days (27.2%) of sample collection. The timings of TV treatment initiation and TV-AE reporting were similar at all CRSs. Overall, 99.1% of TV treatment initiations (eThekwini 100% versus Verulam/Isipingo 98.7%, p=0.554) and 100% TV-AE reporting (eThekwini 100% versus Verulam/Isipingo 100%) occurred on the day of testing. In addition, more NG/CT treatments were initiated during scheduled visits at eThekwini CRS (91.8%) compared to Verulam/Isipingo CRSs (62.2%), p<0.001. In contrast, the percentage of TV treatment initiations at scheduled visits was similar across CRSs (eThekwini=100%, Verulam/Isipingo=93.6%, p=0.178).

### Impact of POC versus central laboratory-based testing on time to STI treatment initiation among women

**Figure 1:**
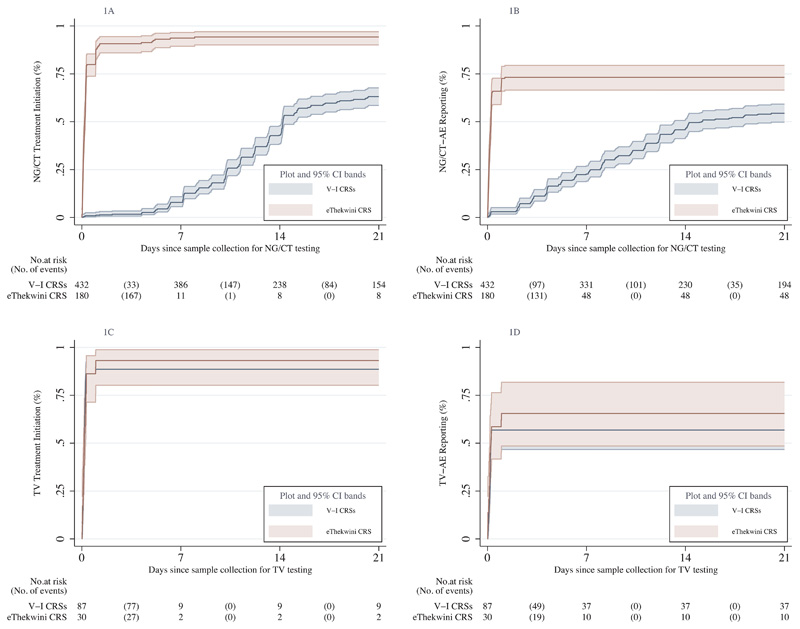
Kaplan-Meier cumulative probability graphs displaying the effects of POC versus central laboratory-based testing on STI treatment initiation and STI-AE reporting after sample collection for testing among women. Figures 1A and 1C compare the time to NG/CT treatment initiation and NG/CT-AE reporting between CRSs that used either POC (eThekwini) or central laboratory-based testing (Verulam and Isipingo). Figures 1B and 1D compare the same groups of CRSs on the time to TV treatment initiation and TV-AE reporting tested with POC assays at all CRSs. Median time in days (95%CI), log-rank test p-value: Figure 1A (V-I CRSs=14.22 (14.12-15.09), eThekwini CRS NG/CT CRS= 0.20 (0.16-0.25), p<0.001). Figure 1B (V-I CRSs=15.12 (13.22-21.24), eThekwini CRS=0.24 (0.19-0.27), p<0.001). Figure 1C (V-I CRS=0.18 (0.15-0.20), eThekwini CRS=0.17 (0.12-0.27), p=0.704). Figure 1D (V-I CRSs=0.24 (0.15-0.99), eThekwini CRS=0.25 (0.20-0.99), p=0.388). V-I CRS, Verulam and Isipingo Clinical Research Sites; NG, *Neisseria gonorrhoeae;* CT, *Chlamydia trachomatis;* TV, *Trichomonas vaginalis;* AE, Adverse Event; POC, Point-of-Care.

The impact of POC testing compared to central laboratory-based testing on trends in the cumulative probability of STI treatment initiation in women is shown in Figures 1A and 1C. Over 9781.0 person-days (PDs) of observation, 572 NG/CT treatments were initiated for 612 NG/CT cases (40 received no treatment in the trial due to lost-to-follow-up). Figure 1A illustrates that NG/CT treatment initiation occurred faster at eThekwini (median days 0.20, 95%CI 0.16-0.25) compared to Verulam/Isipingo CRSs (median days 14.22, 95%CI 14.12-15.09) (p<0.001). In contrast, Figure 1C displays a near-perfect overlap in time to TV treatment initiation with 0.17 (95%CI 0.12-0.27) median days at eThekwini CRS versus 0.18 (95%CI 0.15-0.20) at Verulam/Isipingo CRSs (p=0.704).

Based on the Cox proportional hazard regression results in Table 3, the time to NG/CT treatment initiation was 39 times faster with POC testing at eThekwini CRS compared to laboratory-based testing at Verulam/Isipingo CRSs (aHR=39.62, 95%CI 15.13-103.74, p<0.001), while there was no difference in the time to TV treatment initiation across the CRSs (aHR=0.93, 95%CI 0.59-1.48, p=0.770) after adjusting for demographic variables and study visit type. Older women (>=35 years) compared to younger women (18-24 years) were more likely to receive NG/CT treatment (aHR=1.51, 95%CI 1.12-2.06, p=0.008).

### Impact of POC versus central laboratory-based testing on time to STI-AE reporting among women

The impact of POC compared to central laboratory-based testing on STI-AE reporting trends among women is illustrated in Figures 1B and 1D. Of the 612 NG/CT cases, 404 NG/CT-AEs were reported during 28487.7 PDs of observation (208 were not confirmed as AEs). Figure 1B shows that the trend in NG/CT-AE reporting was faster at eThekwini CRS (median days 0.24, 95%CI 0.19-0.27) compared to Verulam/Isipingo CRSs (median days 15.12, 95%CI 13.22-21.24) (p<0.001). In contrast, Figure 1D shows no difference in TV-AE reporting trends between eThekwini CRS (median days 0.25, 95%CI 0.20-0.99) and Verulam/Isipingo CRSs (median days 0.24, 95%CI 0.15-0.99), p=0.388.

The Cox proportional hazard regression in Table 4 shows that NG/CT-AE reporting was 3.38 times faster with POC testing at eThekwini CRS compared to laboratory-based testing at Verulam/Isipingo CRSs (aHR=3.38, 95%CI 2.23-5.13, p<0.001), while there was no significant difference in the time to TV-AE reporting (aHR=1.38, 95%CI 0.86-2.21, p=0.183) between CRSs, after adjusting for demographic variables and study visit type.

### Sensitivity analysis with disaggregated CRSs and analysis in men

The Cox regression sensitivity analysis of disaggregated CRSs showed identical outcomes between Verulam and Isipingo CRSs (Supplementary Tables 1 and 2). Furthermore, supplementary analysis among men for CT/NG treatment initiation and AE reporting showed identical findings as in women (Supplementary Tables 3-6 and Supplementary Figure 1).

## Discussion

This study tested the hypothesis that POC testing leads to prompt STI management compared to centralised laboratory-based testing among participants from higher-risk communities for STIs in South Africa. We found that NG/CT treatments were initiated 39 times faster, and NG/CT-AEs were reported 3.4 times faster in women when POC testing was used. When we compared TV management, the POC testing effects extended to the research clinics that used central laboratory testing for NG/CT but POC testing for TV, indicating that the findings were due to testing modalities such as result turnaround times rather than other procedures or processes at the clinics. The incidence of STIs was consistent with results from another study among women in the KwaZulu-Natal that reported 15 cases per 100 PYs for chlamydia, gonorrhoea, syphilis or trichomoniasis[32]. Therefore, our results highlight the importance of POC testing as a suitable and effective diagnostic care solution that can improve STI care outcomes and reduce disease burden in South Africa and other endemic resource-limited settings.

Some studies in SSA[33, 34] and globally[19, 20] have evaluated the effects of individual testing approaches (POC or central laboratory-based) on prompt STI treatment initiation, usually without reference to a gold standard. Consistent with our findings, a study in South Africa recorded 91.9% same-day STI (CT, NG, TV) treatment initiations with POC testing in women[33]. Another South African study found that despite the availability of 63.9% of CT positive results within 72 hours after sample collection, treatment was only provided for 56% within seven days, 92.2% within 14 days and 97.5% within 28 days[19].

Our study has added evidence by determining the relative effects of the two current aetiological testing approaches on prompt STI management. Based on the argument that the effectiveness of a diagnostic test depends on the likelihood of leading to early and accurate treatment[35], our findings suggest that POC testing is a clinically more effective approach to central laboratory-based testing for prompt STI management. Furthermore, our results underscore additional practical and potential cost implications for STI/HIV research institutions and health system stakeholders that could lead to a re-think of STI testing approaches. Same-day STI management reduced waiting times and return visits by 91.8%, simplified trial conduct and benefited trial participants by preventing unnecessary clinic visits. Extrapolated to a health care system, the POC testing intervention could potentially reduce the burden on primary health care and STI clinics and result in healthcare savings while at the same time improving STI care[24, 36, 37]. Compared to the syndromic management approach, POC testing could also lead to prompt STI management with better diagnostic accuracy, reducing STI burden and related complications, reinfections, drug resistance, and the risk of HIV transmission.

Our study had some limitations. First, the HVTN702 trial population included a large proportion of women (70%)[27], which is why we focused the analysis on women. Nevertheless, the supplementary analysis confirmed almost identical results for men in the study. Second, our study did not retain the randomised design of the HVTN702 trial, as participants were randomised to receive a vaccine and not an STI testing approach. While baseline characteristics were broadly similar, we cannot rule out the effects of unmeasured confounding variables. However, the almost identical results of the early TV management with POC testing at all CRSs and the Cox regression sensitivity analyses that showed no difference in effect sizes between Verulam and Isipingo clinics further strengthen the validity of our findings.

In conclusion, POC testing led to prompt STI management in a trial setting in South Africa, where such evidence is most needed for decision-making. Our study provides strong evidence to consider POC testing when strengthening STI care systems in resource-limited settings. However, cost, waiting times, diagnostic platforms and error rates are essential practical issues when implementing POC testing for STI care, particularly in resource-limited settings. POC assays are not necessarily cheaper[38], but evidence shows that it is relatively cost-effective for STI care compared to standard laboratory-based approaches[22]. Furthermore, depending on the diagnostic platform and the larger volume of testing expected outside a research setting, waiting times could be prolonged with POC testing. Moreover, POC testing can be prone to errors since it is often conducted by non-laboratory staff, and some studies have estimated error rates of up to 0.65%, mostly occurring at the analytical phase of the testing process[39]. Recognising these potential practical challenges, the WHO developed the ASSURED (affordable, sensitive, specific, user-friendly, rapid, equipment-free, delivered) criteria[40] that describe key features of an ideal POC assay. To improve decision-making in resource-limited settings, further evidence is needed on the feasibility and cost-effectiveness of POC testing models for STI care in under-resourced rural and non-trial settings, as the three clinics in this study were well-resourced trial sites in central/urban areas conducive to the effortless implementation of POC testing.

## Supplementary Material

Supplementary Figure

Supplementary Tables

## Figures and Tables

**Table 1:** Baseline demographic characteristics of enrolled women stratified by clinical research site (CRS)

Variable	Category	Total (N=959) % (n)	Verulam/Isipingo ^a^ CRSs (N=699) % (n)	eThekwini ^b^ CRS (N=260) % n)	P value
Age in years	Median	23 (21-26)	23 (21-27)	23 (21-26)	0.040
Age group in years	18-24	60.3 (578/959)	58.7 (410/699)	64.6 (168/260)	0.083
	25-34	31.6 (303/959)	33.6 (235/699)	26.2 (68/260)	
	35+	8.1 (78/959)	7.7 (54/699)	9.2 (24/260)	
School level completed	High school	61.8 (592/958)	61.7 (431/698)	61.9 (161/260)	0.994
	Primary School	37.8 (362/958)	37.8 (264/698)	37.7 (98/260)	
	No schooling	0.4 (4/958)	0.4 (3/698)	0.4 (1/260)	
Married/stable partner	Yes	96.4 (889/922)	97.3 (651/669)	94.1 (238/253)	0.018
Race/Ethnicity	Black	99.6 (955/959)	99.4 (695/699)	100.0 (260/260)	0.474
	White	0.3 (3/959)	0.4 (3/699)	0.0 (0/260)	
	Indian	0.1 (1/959)	0.1 (1/699)	0.0 (0/260)	
*Neisseria gonorrhoeae* (NG)	Prevalence	3.3 (31/954)	3.5 (24/694)	2.7 (7/260)	0.552
*Chlamydia trachomatis* (*CT*)	Prevalence	19.8 (189/956)	20.6 (143/696)	17.7 (46/260)	0.324
NG/CT	Prevalence	21.4 (205/958)	22.4 (156/698)	18.9 (49/260)	0.240
*Trichomonas vaginalis ^c^* (TV)	Prevalence	4.7 (44/946)	5.1 (35/691)	3.5 (9/255)	0.320

Denominators that do not equal sample sizes are due to missing data. Percentages may not total 100 because of rounding.

a Central laboratory-based testing for NG/CT was conducted at the Isipingo and Verulam clinical research sites (CRSs).

b POC testing for NG/CT was conducted at the eThekwini CRS.

c All CRSs used POC assays for TV testing.

**Table 2:** Overall STI tests, incidence, positive cases, treatment initiation and STI-AE reporting stratified by clinical research site (CRS)

Variable	Category	Total (N=959) % (n)	Verulam/Isipingo ^a^ CRSs (N=699) % (n)	eThekwini ^b^ CRS (N=260) % (n)	P-value
*Neisseria gonorrhoeae* (NG)	Incidence ^d^ (new cases/PYs)	6.7 (122/1822.9)	6.2 (80/1305.9)	8.2 (42/517)	0.143
*Chlamydia trachomatis* (CT)	Incidence ^d^ (new cases/PYs)	24.8 (364/1471.5)	25.7 (268/1045.8)	22.6 (96/425.7)	0.283
NG/CT	Incidence ^d^ (new cases/PYs)	28.8 (406/1414.0)	29.7 (298/1005.2)	26.5 (108/408.8)	0.305
Trichomonas vaginalis ^c^ (TV)	Incidence ^d^ (new cases/PYs)	4.9 (89/1847.1)	5.2 (67/1310.7)	4.2 (22/536.4)	0.374
NG	Positive	3.7 (143/3830)	3.4 (91/2692)	4.6 (52/1138)	0.076
CT	Positive	13.5 (518/3828)	13.8 (371/2692)	12.9 (147/1136)	0.487
NG/CT	Positive	16.0 (612/3838)	16.0 (432/2700)	15.8 (180/1138)	0.888
TV	Positive	3.1 (117/3784)	3.3 (87/2656)	2.7 (30/1128)	0.317
NG	Treatments initiated	76.9 (110/143)	80.2 (73/91)	71.2 (37/52)	0. 216
CT	Treatments initiated	94.2 (488/518)	93.3 (346/371)	96.6 (142/147)	0.143
NG/CT	Treatments initiated	93.5 (572/612)	93.1 (402/432)	94.4 (170/180)	0.526
TV	Treatments initiated	89.7 (105/117)	89.7 (78/87)	90.0 (27/30)	0.957
Time to NG treatment initiation after sample collection	Same day	31.8 (35/110)	1.4 (1/73)	91.9 (34/37)	< 0.001
	2-7 days	6.4 (7/110)	6.9 (5/73)	5.4 (2/37)	
	8-14 days	30.9 (34/110)	45.2 (33/73)	2.7 (1/37)	
	After 14 days	30.9 (34/110)	46.6 (34/73)	0.0 (0/37)	
Time to CT treatment initiation after sample collection	Same day	27.9 (136/488)	1.5 (5/346)	92.3 (131/142)	< 0.001
	2-7 days	7.0 (34/488)	7.5 (26/346)	5.6 (8/142)	
	8-14 days	25.2 (123/488)	35.3 (122/346)	0.7 (1/142)	
	After 14 days	40.0 (195/488)	55.8 (193/346)	1.4 (2/142)	
Time to NG/CT treatment initiation after sample collection	Same day	28.3 (162/572)	1.2 (5/402)	92.4 (157/170)	< 0.001
	2-7 days	6.6 (38/572)	7.0 (28/402)	5.9 (10/170)	
	8-14 days	25.9 (148/572)	36.6 (147/402)	0.6 (1/170)	
	After 14 days	39.2 (224/572)	55.2 (222/402)	1.2 (2/170)	
Type of study visit at which NG/CT treatments were initiated	Scheduled	71.0 (406/572)	62.2 (250/402)	91.8 (156/170)	< 0.001
	Unscheduled	29.0 (166/572)	37.8 (152/402)	8.2 (14/170)	
Time to TV treatment initiation after sample collection	Same day	99.1 (104/105)	98.7 (77/78)	100.0 (27/27)	0.554
	2-7 days	0.0 (0/0)	0.0 (0/0)	0.0 (0/0)	
	8-14 days	1.0 (1/105)	1.3 (1/78)	0.0 (0/27)	
	After 14 days	0.0 (0/0)	0.0 (0/0)	0.0 (0/0)	
Type of study visit at which TV treatments were initiated	Scheduled	95.2 (100/105)	93.6 (73/78)	100.0 (27/27)	0.178
	Unscheduled	4.8 (5/105)	6.4 (5/78)	0.0 (0/27)	
NG-AEs reported	Yes	72.0 (103/143)	67.0 (61/91)	80.8 (42/52)	0.078
CT-AEs reported	Yes	65.4 (339/518)	63.3 (235/371)	70.8 (104/147)	0.110
NG/CT-AEs reported	Yes	66.0 (404/612)	63.0 (272/432)	73.3 (132/180)	0.014
TV-AEs reported	Yes	58.1 (68/117)	56.3 (49/87)	63.3 (19/30)	0.502
Time to NG-AE reporting after sample collection	Same day	44.7 (46/103)	8.2 (5/61)	97.6 (41/42)	< 0.001
	2-7 days	15.5 (16/103)	24.6 (15/61)	2.4 (1/42)	
	8-14 days	25.2 (26/103)	42.6 (26/61)	0.0 (0/42)	
	After 14 days	14.6 (15/103)	24.6 (15/61)	0.0 (0/42)	
Time to CT-AE reporting after sample collection	Same day	36.6 (124/339)	8.5 (20/235)	100 (104/104)	< 0.001
	2-7 days	22.4 (76/339)	32.3 (76/235)	0.0 (0/104)	
	8-14 days	23.9 (81/339)	34.5 (81/235)	0.0 (0/104)	
	After 14 days	17.1 (58/339)	24.7 (58/235)	0.0 (0/104)	
Time to NG/CT-AE reporting after sample collection	Same day	35.4 (143/404)	4.8 (13/272)	98.5 (130/132)	< 0.001
	2-7 days	21.0 (85/404)	30.9 (84/272)	0.8 (1/132)	
	8-14 days	25.0 (101/404)	37.1 (101/272)	0.0 (0/132)	
	After 14 days	18.6 (75/404)	27.2 (74/272)	0.8 (1/132)	
Type of study visit at which	Scheduled	97.5 (394/404)	97.1 (264/272)	98.5 (130/132)	0.387
NG/CT-AEs were reported	Unscheduled	2.5 (10/404)	2.9 (8/272)	1.5 (2/132)	
Time to TV-AE reporting after sample collection	Same day	100.0 (68/68)	100.0 (49/49)	100.0 (19/19)	-
	2-7 days	-	-	-	
	8-14 days	-	-	-	
	After 14 days	-	-	-	
Type of study visit at which TV-	Scheduled	98.5 (67/68)	98.0 (48/49)	100 (19/19)	0.530
AEs were reported	Unscheduled	1.5 (1/68)	2.0 (1/49)	0.0 (0/19)	

Denominators that do not equal sample sizes are due to missing data. Percentages may not total 100 because of rounding. STI, Sexually Transmitted Infection; AE, Adverse Event; POC, Point-of-Care; N= number of women, n= number of observations during followup including repeat testing.

a Central laboratory-based testing for NG/CT was conducted at the Isipingo and Verulam clinical research sites (CRSs).

b POC testing for NG/CT was conducted at the eThekwini CRS.

c All CRSs used POC assays for TV testing.

d Incidence was calculated as the number of new cases per 100 person-years (PYs).

**Table 3:** Cox proportional hazard regression for the impact of POC versus central laboratory-based testing on time to STI treatment initiation among women

Variable	Category	Model A. NG/CT outcome			Model B.TV ^c^ outcome		
		Treatments/ Person Days	aHR (95%CI)	P-value	Treatments/ Person Days	aHR (95%CI)	P-value
Age group in years	18-24	413/8913.7	1	-	55/1312.2	1	-
	25-34	138/3080.6	1.02 (0.83-1.25)	0.840	37/174.9	1.48 (0.96-2.29)	0.075
	35+	21/230.4	1.51 (1.12-2.06)	0.008	13/4.4	1.05 (0.65-1.68)	0.844
School level completed	High school	354/7786.9	1	-	59/1208.1	1	-
	Primary school	216/4341.4	1.16 (0.45-2.98)	0.753	44/460.3	0.99 (0.66-1.47)	0.955
	No school	2/96.4	1.21 (0.47-3.13)	0.689	2/0.3	0.96 (0.61-1.51)	0.863
CRS ^d^	Verulam/Isipingo ^a^	402/11396.3	1	-	78/1124.0	1	-
	eThekwini ^b^	170/828.3	39.62 (15.13-103.74)	< 0.001	27/534.8	0.93 (0.59-1.48)	0.770
Study visit type at which STI treatments were initiated ^d^	Scheduled	406/9430.6	1	-	100/1634.9	1	-
	Unscheduled	166/2794.1	0.76 (0.52-1.11)	0.152	5/33.9	0.71 (0.38-1.32)	0.281

Denominators that do not equal the sample sizes are due to missing data. STI, Sexually Transmitted Infection; CT, *Chlamydia trachomatis;* NG, *Neisseria gonorrhoeae;* TV, *Trichomonas vaginalis;* CRS, Clinical research Site; POC, Point-of-Care; aHR, adjusted Hazard Ratio; CI, Confidence Interval.

a Central laboratory-based testing for NG/CT was conducted at the Isipingo and Verulam clinical research sites (CRSs).

b POC testing for NG/CT was conducted at the eThekwini CRS.

c All CRSs used POC assays for TV testing.

d Variable specified as a time-varying-covariate in Model A to satisfy proportional hazard assumption. Model B satisfied the proportional hazard assumption (Schoenfeld test p=0.1570).

**Table 4:** Cox proportional hazard regression for the impact of POC versus central laboratory-based testing on time to STI-AE reporting among women

Variable	Category	**Model A.** NG/CT outcome			**Model B.** TV ^c^ outcome		
		AEs reported/ Person Days	aHR (95%CI)	P-value	AEs reported/ Person Days	aHR (95%CI)	P-value
Age group in years	18-24	302/19938.0	1	-	34/3567.4	1	-
	25-34	94/7049.9	0.96 (0.77-1.2)	0.705	26/1453.8	1.53 (0.96-2.43)	0.074
	35+	8/1499.9	0.46 (0.22-0.95)	0.035	8/820.9	0.95 (0.52-1.73)	0.871
School level completed	High school	249/17610.0	1	-	37/3705.0	1	-
	Primary school	154/10730.4	1.84 (0.43-7.93)	0.413	30/2096.3	1.79 (1.12-2.86)	0.014
	No school	1/147.4	1.91 (0.44-8.22)	0.386	1/113.3	2.00 (1.36-2.93)	< 0.001
CRS ^d^	Verulam/Isipingo ^a^	272/22453.1	1	-	49/4384.0	1	-
	eThekwini ^b^	132/6034.5	3.38 (2.23-5.13)	< 0.001	19/1461.8	1.38 (0.86-2.21)	0.183
Study visit type at which	Scheduled	394/28346.2	1	-	67/5914.4	1	-
STI-AEs were reported	Unscheduled	10/141.6	1.76 (1.22-2.54)	0.002	1/0.3	0.99 (0.67-1.46)	0.960

Denominators that do not equal the sample sizes are due to missing data. STI, Sexually Transmitted Infection; CT, *Chlamydia trachomatis*; NG, *Neisseria gonorrhoeae*; TV, *Trichomonas vaginalis*; CRS, Clinical research Site; POC, Point-of-Care; AE, Adverse Event; aHR, adjusted Hazard Ratio; CI, Confidence Interval.

a Central laboratory-based testing for NG/CT was conducted at the Isipingo and Verulam clinical research sites (CRSs).

b POC testing for NG/CT was conducted at the eThekwini CRS.

c All CRSs used POC assays for TV testing.

d Variable specified as a time-varying-covariate in Model A to satisfy proportional hazard assumption. Model B satisfied the proportional hazard assumption (Schoenfeld test p=0.4881).

## Summary

We compared the impact of point-of-care versus centralised laboratory-based testing on managing sexually transmitted infections among HVTN702 vaccine trial participants in South Africa. Point-of-care testing led to faster STI diagnosis, treatment initiation and reporting of adverse events that were STIs.

## References

[1] World Health Organisation GUIDELINES FOR THE MANAGEMENT OF SEXUALLY TRANSMITTED INFECTIONS.

[2] Centers for Disease Control and Prevention Detailed STD Facts - Gonorrhea.

[3] Centers for Disease Control and Prevention Detailed STD Facts - Chlamydia.

[4] Centers for Disease Control and Prevention Trichomoniasis - CDC Fact Sheet.

[5] WHO (2016). Global health sector strategy on Sexually Transmitted Infections 2016-2021.

[6] Weström L, Joesoef R, Reynolds G, Hagdu A, Thompson SE (1992). Pelvic inflammatory disease and fertility. A cohort study of 1,844 women with laparoscopically verified disease and 657 control women with normal laparoscopic results. Sex Transm Dis.

[7] Centers for Disease Control and Prevention Syphilis - CDC Fact Sheet (Detailed).

[8] Kaida A, Dietrich JJ, Laher F (2018). A high burden of asymptomatic genital tract infections undermines the syndromic management approach among adolescents and young adults in South Africa: implications for HIV prevention efforts. BMC Infect Dis.

[9] Centres for Disease Control and Prevention STDs and HIV – CDC Fact Sheet.

[10] Garrett NJ, Osman F, Maharaj B (2018). Beyond syndromic management: Opportunities for diagnosis-based treatment of sexually transmitted infections in low- and middle-income countries. PLOS ONE.

[11] Garrett NJ, McGrath N, Mindel A (2017). Advancing STI care in low/middle-income countries: has STI syndromic management reached its use-by date?. Sexually Transmitted Infections.

[12] Mlisana K, Naicker N, Werner L (2012). Symptomatic Vaginal Discharge Is a Poor Predictor of Sexually Transmitted Infections and Genital Tract Inflammation in High-Risk Women in South Africa. The Journal of Infectious Diseases.

[13] Hawkes S, Morison L, Foster S (1999). Reproductive-tract infections in women in low-income, low-prevalence situations: assessment of syndromic management in Matlab, Bangladesh. Lancet.

[14] Barry MS, Ba Diallo A, Diadhiou M (2018). Accuracy of syndromic management in targeting vaginal and cervical infections among symptomatic women of reproductive age attending primary care clinics in Dakar, Senegal. Tropical Medicine & International Health.

[15] Tann CJ, Mpairwe H, Morison L (2006). Lack of effectiveness of syndromic management in targeting vaginal infections in pregnancy in Entebbe, Uganda. Sex Transm Infect.

[16] Ong JJ, Magooa MP, Chikandiwa A (2019). Clinical Characteristics of Mycoplasma genitalium and the Usefulness of Syndromic Management Among Women Living With Human Immunodeficiency Virus. Sex Transm Dis.

[17] World Health Organisation Sexually transmitted infections (STIs).

[18] Vargas S, Calvo G, Qquellon J (2022). Point-of-care testing for sexually transmitted infections in low-resource settings. Clinical microbiology and infection : the official publication of the European Society of Clinical Microbiology and Infectious Diseases.

[19] Proctor G, Sarner L, Symonds M (2017). P182 Chlamydia positive testing to treatment turnaround time (TAT) april to december 2016. Sexually Transmitted Infections.

[20] Geisler WM, Wang C, Morrison SG, Black CM, Bandea CI, Hook EW (2008). The natural history of untreated Chlamydia trachomatis infection in the interval between screening and returning for treatment. Sex Transm Dis.

[21] Drain PK, Garrett NJ (2015). The arrival of a true point-of-care molecular assay—ready for global implementation?. The Lancet Global Health.

[22] Huntington SE, Burns RM, Harding-Esch E (2018). Modelling-based evaluation of the costs, benefits and cost-effectiveness of multipathogen point-of-care tests for sexually transmitted infections in symptomatic genitourinary medicine clinic attendees. BMJ Open.

[23] Garrett N, Mtshali A, Osman F (2021). Impact of point-of-care testing and treatment of sexually transmitted infections and bacterial vaginosis on genital tract inflammatory cytokines in a cohort of young South African women. Sexually transmitted infections.

[24] Garrett N, Mitchev N, Osman F (2019). Diagnostic accuracy of the Xpert CT/NG and OSOM Trichomonas Rapid assays for point-of-care STI testing among young women in South Africa: a cross-sectional study. BMJ Open.

[25] Negash M, Wondmagegn T, Geremew D (2018). Comparison of RPR and ELISA with TPHA for the Diagnosis of Syphilis: Implication for Updating Syphilis Point-of-Care Tests in Ethiopia. Journal of Immunology Research.

[26] Gaydos CA, Klausner JD, Pai NP, Kelly H, Coltart C, Peeling RW (2017). Rapid and point-of-care tests for the diagnosis of Trichomonas vaginalis in women and men. Sexually Transmitted Infections.

[27] Gray GE, Bekker LG, Laher F (2021). Vaccine Efficacy of ALVAC-HIV and Bivalent Subtype C gp120-MF59 in Adults. N Engl J Med.

[28] Stata Corporation (2017). Stata statistical software release 17.

[29] Hertz-Picciotto I, Rockhill B (1997). Validity and Efficiency of Approximation Methods for Tied Survival Times in Cox Regression. Biometrics.

[30] Zhang Z, Reinikainen J, Adeleke KA, Pieterse ME, Groothuis-Oudshoorn CGM (2018). Time-varying covariates and coefficients in Cox regression models. Annals of Translational Medicine.

[31] Kuitunen I, Ponkilainen VT, Uimonen MM, Eskelinen A, Reito A (2021). Testing the proportional hazards assumption in cox regression and dealing with possible non-proportionality in total joint arthroplasty research: methodological perspectives and review. BMC Musculoskeletal Disorders.

[32] Wand H, Reddy T, Dassaye R, Moodley J, Naidoo S, Ramjee G (2020). Estimating prevalence and incidence of sexually transmitted infections among South African women: Implications of combined impacts of risk factors. International Journal of STD & AIDS.

[33] Morikawa E, Mudau M, Olivier D (2018). Acceptability and Feasibility of Integrating Point-of-Care Diagnostic Testing of Sexually Transmitted Infections into a South African Antenatal Care Program for HIV-Infected Pregnant Women. Infectious Diseases in Obstetrics and Gynecology.

[34] Myer L (2003). Impact of on-site testing for maternal syphilis on treatment delays, treatment rates, and perinatal mortality in rural South Africa: a randomised controlled trial. Sexually Transmitted Infections.

[35] Price CP (2001). Point of care testing. BMJ (Clinical research ed).

[36] Huang W, Gaydos CA, Barnes MR, Jett-Goheen M, Blake DR (2013). Comparative effectiveness of a rapid point-of-care test for detection of Chlamydia trachomatis among women in a clinical setting. Sexually Transmitted Infections.

[37] Huppert J, Hesse E, Gaydos CA (2010). What is the point? How point-of-care sexually transmitted infection tests can impact infected patients. Point of Care.

[38] Simeon K, Sharma M, Dorward J (2019). Comparative cost analysis of point-of-care versus laboratory-based testing to initiate and monitor HIV treatment in South Africa. PLOS ONE.

[39] O’Kane MJ, McManus P, McGowan N, Lynch PM (2011). Quality Error Rates in Point-of-Care Testing. Clinical Chemistry.

[40] Kettler H, White K, Hawkes SJ, Research UNWBWSPf, Training in Tropical D Mapping the landscape of diagnostics for sexually transmitted infections : key findings and recommendations / Hannah Kettler, Karen White, Sarah Hawkes.

