# Supplementary material for: Impact of point-of-care testing on the management of sexually transmitted infections in South Africa: Evidence from the HVTN702 HIV vaccine trial
Source: Clin Infect Dis. Author manuscript; Available in PMC 2023 Mar 9. (PMC7614294; doi:10.1093/cid/ciac824)
Supplement: Supplementary Figure [file EMS157961-supplement-Supplementary_Figure.pdf]

Supplementary Figure 1A

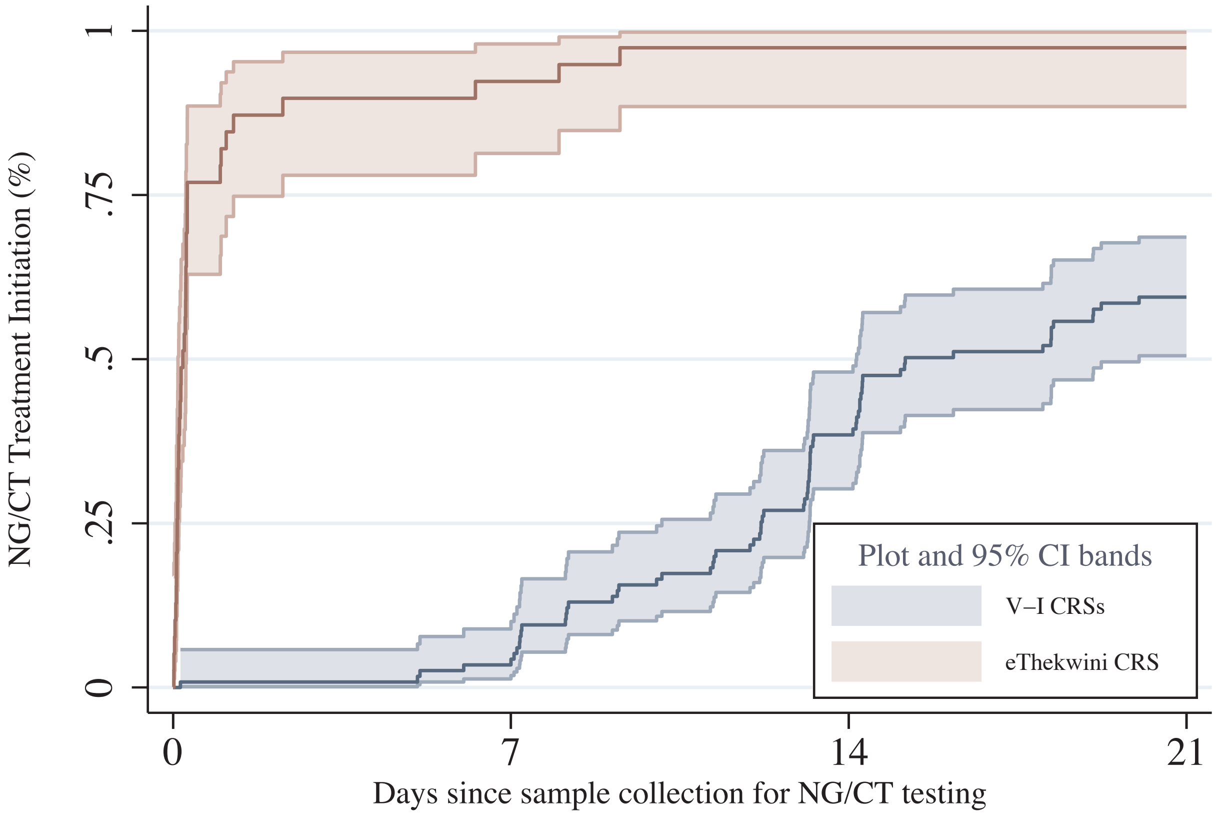

No.at risk (No. of events)							
V–I CRSs	121	(4)	111	(40)	69	(23)	44
eThekwini CRS	39	(36)	3	(2)	1	(0)	1

Supplementary Figure 1B

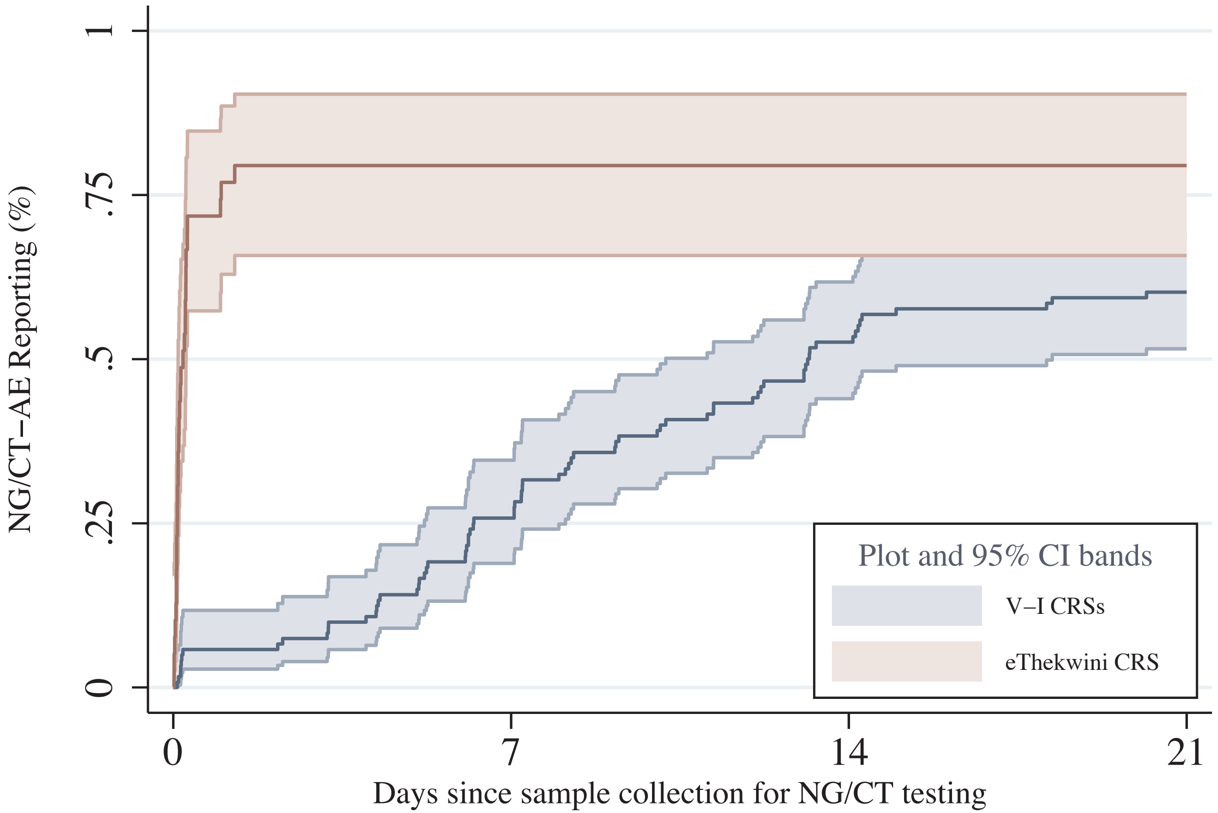

No.at risk (No. of events)							
V–I CRSs	121	(31)	89	(32)	56	(9)	47
eThekwini CRS	39	(31)	8	(0)	8	(0)	8
