# Supplementary material for: Impact of point-of-care testing on the management of sexually transmitted infections in South Africa: Evidence from the HVTN702 HIV vaccine trial
Source: Clin Infect Dis. Author manuscript; Available in PMC 2023 Mar 9. (PMC7614294; doi:10.1093/cid/ciac824)
Supplement: Supplementary Tables [file EMS157961-supplement-Supplementary_Tables.docx]

| **Supplementary Table 1.** Sensitivity analysis with disaggregated CRSs. Cox proportional hazard regression for the impact of POC versus central laboratory-based testing on time to STI treatment initiation among women | | | | | | | | | |
| --- | --- | --- | --- | --- | --- | --- | --- | --- | --- |
|  |  |  | **Model A.** NG/CT outcome | | |  | **Model B.** TV ^c^ outcome | | |
| Variable | Category |  | Treatments/  Person Days | aHR (95%CI) | P-value |  | Treatments/  Person Days | aHR (95%CI) | P-value |
| Age group in years | 18-24 |  | 413/8913.7 | 1 | - |  | 55/1312.2 | 1 | - |
|  | 25-34 |  | 138/3080.6 | 1.02 (0.83-1.25) | 0.826 |  | 37/174.9 | 1.62 (1.04-2.52) | 0.031 |
|  | 35+ |  | 21/230.4 | 1.52 (1.12-2.06) | 0.008 |  | 13/4.4 | 1.09 (0.66-1.81) | 0.736 |
| School level completed | High school |  | 354/7786.9 | 1 | - |  | 59/1208.1 | 1 | - |
|  | Primary school |  | 216/4341.4 | 1.18 (0.45-3.05) | 0.741 |  | 44/460.3 | 0.86 (0.55-1.34) | 0.500 |
|  | No school |  | 2/96.4 | 1.23 (0.47-3.21) | 0.676 |  | 2/0.3 | 0.87 (0.55-1.37) | 0.538 |
| CRS ^d^ | Isipingo ^a^ |  | 205/ 5898.2 | 1 | - |  | 36/454.7 | 1 | - |
|  | Verulam ^a^ |  | 197/ 5498.1 | 1.02 (0.84-1.25) | 0.809 |  | 42/648.2 | 1.38 (0.81-2.35) | 0.232 |
|  | eThekwini ^b^ |  | 170/828.3 | 40.10 (15.37-104.63) | < 0.001 |  | 27/534.8 | 1.13 (0.67-1.91) | 0.656 |
| Study visit type at which STI treatments were initiated ^d^ | Scheduled |  | 406/9430.6 | 1 | - |  | 100/1634.9 | 1 | - |
|  | Unscheduled |  | 166/2794.1 | 0.76 (0.52-1.11) | 0.152 |  | 5/33.9 | 0.82 (0.42-1.59) | 0.556 |

Denominators that do not equal the sample sizes are due to missing data.

STI, Sexually Transmitted Infection; CT, *Chlamydia trachomatis*; NG, *Neisseria gonorrhoeae*; TV, *Trichomonas vaginalis*; CRS, Clinical research Site;

POC, Point-of-Care; aHR, adjusted Hazard Ratio; CI, Confidence Interval.

^a^ Central laboratory-based testing for NG/CT was conducted at the Isipingo and Verulam clinical research sites (CRSs).

^b^ POC testing for NG/CT was conducted at the eThekwini CRS.

^c^ All CRSs used POC assays for TV testing.

^d^ Variable specified as a time-varying-covariate in Model A to satisfy proportional hazard assumption. Model B satisfied the proportional hazard

assumption (Schoenfeld test p=0.2501).

| **Supplementary Table 2.** Sensitivity analysis with disaggregated CRSs. Cox proportional hazard regression for the impact of POC versus central laboratory-based testing on time to STI-AE reporting among women | | | | | | | | | |
| --- | --- | --- | --- | --- | --- | --- | --- | --- | --- |
|  |  |  | **Model A.** NG/CT outcome | | |  | **Model B.** TV ^c^ outcome | | |
| Variable | Category |  | AEs reported/  Person Days | aHR (95%CI) | P-value |  | AEs reported /  Person Days | aHR (95%CI) | P-value |
| Age group in years | 18-24 |  | 302/19938.0 | 1 | - |  | 34/3567.4 | 1 | - |
|  | 25-34 |  | 94/7049.9 | 0.95 (0.76-1.19) | 0.680 |  | 26/1453.8 | 1.58 (0.95-2.62) | 0.079 |
|  | 35+ |  | 8/1499.9 | 0.45 (0.22-0.94) | 0.034 |  | 8/820.9 | 0.97 (0.53-1.8) | 0.932 |
| School level completed | High school |  | 249/17610.0 | 1 | - |  | 37/3705.0 | 1 | - |
|  | Primary school |  | 154/10730.4 | 1.84 (0.41-8.33) | 0.427 |  | 30/2096.3 | 1.71 (1.03-2.84) | 0.039 |
|  | No school |  | 1/147.4 | 1.90 (0.42-8.6) | 0.404 |  | 1/113.3 | 1.91 (1.29-2.82) | 0.001 |
| CRS ^d^ | Isipingo ^a^ |  | 137/ 11291.3 | 1 | - |  | 23/1908.6 | 1 | - |
|  | Verulam ^a^ |  | 135/ 11161.8 | 0.93 (0.74-1.17) | 0.544 |  | 26/2475.4 | 1.13 (0.66-1.93) | 0.663 |
|  | eThekwini ^b^ |  | 132/6034.5 | 3.26 (2.1-5.08) | < 0.001 |  | 19/1461.8 | 1.47 (0.86-2.52) | 0.157 |
| Study visit type at which STI-AEs were reported | Scheduled |  | 394/28346.2 | 1 | - |  | 67/5914.4 | 1 | - |
|  | Unscheduled |  | 10/141.6 | 1.79 (1.24-2.57) | 0.002 |  | 1/0.3 | 1.03 (0.7-1.54) | 0.866 |

Denominators that do not equal the sample sizes are due to missing data.

STI, Sexually Transmitted Infection; CT, *Chlamydia trachomatis*; NG, *Neisseria gonorrhoeae*; TV, *Trichomonas vaginalis*; CRS, Clinical research Site;

POC, Point-of-Care; AE, Adverse Event; aHR, adjusted Hazard Ratio; CI, Confidence Interval.

^a^ Central laboratory-based testing for NG/CT was conducted at the Isipingo and Verulam clinical research sites (CRSs).

^b^ POC testing for NG/CT was conducted at the eThekwini CRS.

^c^ All CRSs used POC assays for TV testing.

^d^ Variable specified as a time-varying-covariate in Model A to satisfy proportional hazard assumption. Model B satisfied the proportional hazard

assumption (Schoenfeld test p=0.5851).

| **Supplementary Table 3.** Baseline demographic characteristics of enrolled men stratified by clinical research site (CRS) | | | | | | |
| --- | --- | --- | --- | --- | --- | --- |
| Variable | Category |  | Total  (N=396)  % (n) | Verulam/Isipingo ^a^ CRSs (N=301)  % (n) | eThekwini ^b^ CRS  (N=95)  % (n) | P value |
| Age in years | Median |  | 23 (21-27) | 23 (21-26) | 26 (23-28) | < 0.001 |
| Age group in years | 18-24 |  | 58.1 (230/396) | 63.1 (190/301) | 42.1 (40/95) | 0.001 |
|  | 25-34 |  | 30.3 (120/396) | 26.9 (81/301) | 41.1 (39/95) |  |
|  | 35+ |  | 11.6 (46/396) | 10.0 (30/301) | 16.8 (16/95) |  |
| School level completed | High school |  | 56.3 (223/396) | 58.8 (177/301) | 48.4 (46/95) | 0.170 |
|  | Primary School |  | 42.4 (168/396) | 40.2 (121/301) | 49.5 (47/95) |  |
|  | No schooling |  | 1.3 (5/396) | 1.0 (3/301) | 2.1 (2/95) |  |
| Married/stable partner | Yes |  | 93.5 (359/384) | 95.9 (279/291) | 86.0 (80/93) | 0.001 |
| Race/Ethnicity | Black |  | 100 (396/396) | 100 (301/301) | 100 (95/95) | - |
|  | White |  | - | - | - |  |
|  | Indian |  | - | - | - |  |
| *Neisseria gonorrhoeae* (NG) | Prevalence |  | 1.0 (4/396) | 1.0 (3/301) | 1.1 (1/95) | 0.869 |
| *Chlamydia trachomatis (CT)* | Prevalence |  | 12.7 (50/393) | 13.0 (39/300) | 11.8 (11/93) | 0.767 |
| NG/CT | Prevalence |  | 13.1 (52/396) | 13.3 (40/301) | 12.6 (12/95) | 0.869 |

Denominators that do not equal sample sizes are due to missing data. Percentages may not total 100 because of rounding.

^a^ Central laboratory-based testing for NG/CT was conducted at the Isipingo and Verulam clinical research sites (CRSs).

^b^ POC testing for NG/CT was conducted at the eThekwini CRS.

| **Supplementary Table 4.** Overall STI tests, incidence, positive cases, treatment initiation and STI-AE reporting among men stratified by clinical research site (CRS) | | | | | | |
| --- | --- | --- | --- | --- | --- | --- |
| Variable | Category |  | Total  (N=396)  % (n) | Verulam/Isipingo ^a^ CRSs (N=301)  % (n) | eThekwini ^b^ CRS (N=95)  % (n) | P-value |
| *Neisseria gonorrhoeae* (NG) | Incidence ^c^ (new cases/PYs) |  | 2.4 (20/865.9) | 2.5 (16/663.2) | 2.0 (4/202.7) | 0.758 |
| *Chlamydia trachomatis* (CT) | Incidence ^c^ (new cases/PYs) |  | 10.6 (77/732.4) | 10.2 (57/560.8) | 11.7 (20/171.6) | 0.590 |
| NG/CT | Incidence ^c^ (new cases/PYs) |  | 12.1 (87/724.2) | 11.5 (64/556.7) | 13.8 (23/167.4) | 0.461 |
| NG | Positive |  | 1.4 (25/1772) | 1.5 (20/1329) | 1.1 (5/443) | 0.561 |
| CT | Positive |  | 8.2 (145/1759) | 8.4 (111/1318) | 7.7 (34/441) | 0.638 |
| NG/CT | Positive |  | 9.0 (160/1774) | 9.1 (121/1330) | 8.8 (39/444) | 0.842 |
| TV | Treatments initiated |  | 72.0 (18/25) | 65.0 (13/20) | 100 (5/5) | 0.119 |
| CT | Treatments initiated |  | 91.0 (132/145) | 89.2 (99/111) | 97.1 (33/34) | 0.160 |
| NG/CT | Treatments initiated |  | 90.6 (145/160) | 88.4 (107/121) | 97.4 (38/39) | 0.093 |
| Time to NG treatment initiation after sample collection | Same day |  | 27.8 (5/18) | 7.7 (1/13) | 80.0 (4/5) | 0.003 |
|  | 2-7 days |  | 5.6 (1/18) | 0.0 (0/13) | 20.0 (1/5) |  |
|  | 8-14 days |  | 22.2 (4/18) | 30.8 (4/13) | 0.0 (0/5) |  |
|  | After 14 days |  | 44.4 (8/18) | 61.5 (8/13) | 0.0 (0/5) |  |
| Time to CT treatment initiation after sample collection | Same day |  | 22.0 (29/132) | 1.0 (1/99) | 84.9 (28/33) | < 0.001 |
|  | 2-7 days |  | 4.6 (6/132) | 3.0 (3/99) | 9.1 (3/33) |  |
|  | 8-14 days |  | 30.3 (40/132) | 38.4 (38/99) | 6.1 (2/33) |  |
|  | After 14 days |  | 43.2 (57/132) | 57.6 (57/99) | 0.0 (0/33) |  |
| Time to NG/CT treatment initiation after sample collection | Same day |  | 22.8 (33/145) | 0.9 (1/107) | 84.2 (32/38) | < 0.001 |
|  | 2-7 days |  | 4.8 (7/145) | 2.8 (3/107) | 10.5 (4/38) |  |
|  | 8-14 days |  | 29.0 (42/145) | 37.4 (40/107) | 5.3 (2/38) |  |
|  | After 14 days |  | 43.5 (63/145) | 58.9 (63/107) | 0.0 (0/38) |  |
| Type of study visit at which NG/CT treatments were initiated | Scheduled |  | 69.0 (100/145) | 65.4 (70/107) | 79.0 (30/38) | 0.122 |
|  | Unscheduled |  | 31.0 (45/145) | 34.6 (37/107) | 21.1 (8/38) |  |
| NG-AEs reported | Yes |  | 88.0 (22/25) | 85.0 (17/20) | 100 (5/5) | 0.356 |
| CT-AEs reported | Yes |  | 71.0 (103/145) | 69.4 (77/111) | 76.5 (26/34) | 0.424 |
| NG/CT-AEs reported | Yes |  | 72.5 (116/160) | 70.3 (85/121) | 79.5 (31/39) | 0.261 |
| Time to NG-AE reporting after sample collection | Same day |  | 40.9 (9/22) | 23.5 (4/17) | 100 (5/5) | 0.025 |
|  | 2-7 days |  | 9.1 (2/22) | 11.8 (2/17) | 0.0 (0/5) |  |
|  | 8-14 days |  | 27.3 (6/22) | 35.3 (6/17) | 0.0 (0/5) |  |
|  | After 14 days |  | 22.7 (5/22) | 29.4 (5/17) | 0.0 (0/5) |  |
| Time to CT-AE reporting after sample collection | Same day |  | 29.1 (30/103) | 6.5 (5/77) | 96.2 (25/26) | < 0.001 |
|  | 2-7 days |  | 22.3 (23/103) | 28.6 (22/77) | 3.9 (1/26) |  |
|  | 8-14 days |  | 28.2 (29/103) | 37.7 (29/77) | 0.0 (0/26) |  |
|  | After 14 days |  | 20.4 (21/103) | 27.3 (21/77) | 0.0 (0/26) |  |
| Time to NG/CT-AE reporting after sample collection | Same day |  | 31.9 (37/116) | 8.2 (7/85) | 96.8 (30/31) | < 0.001 |
|  | 2-7 days |  | 21.6 (25/116) | 28.2 (24/85) | 3.2 (1/31) |  |
|  | 8-14 days |  | 27.6 (32/116) | 37.7 (32/85) | 0.0 (0/31) |  |
|  | After 14 days |  | 19.0 (22/116) | 25.9 (22/85) | 0.0 (0/31) |  |
| Type of study visit at which NG/CT-AEs were reported | Scheduled |  | 95.7 (111/116) | 97.7 (83/85) | 90.3 (28/31) | 0.086 |
|  | Unscheduled |  | 4.3 (5/116) | 2.4 (2/85) | 9.7 (3/31) |  |

Denominators that do not equal sample sizes are due to missing data. Percentages may not total 100 because of rounding.

STI, Sexually Transmitted Infection; AE, Adverse Event; POC, Point-of-Care; N= number of women, n= number of observations during follow-up including repeat testing.

^a^ Central laboratory-based testing for NG/CT was conducted at the Isipingo and Verulam clinical research sites (CRSs).

^b^ POC testing for NG/CT was conducted at the eThekwini CRS.

^c^ Incidence was calculated as the number of new cases per 100 person-years (PYs).

| **Supplementary Table 5.** Cox proportional hazard regression for the impact of POC versus central laboratory-based testing on time to (A) NG/CT treatment initiation and (B) NG/CT-AE reporting among men | | | | | | | | | |
| --- | --- | --- | --- | --- | --- | --- | --- | --- | --- |
|  |  |  | **Model A.** Time to NG/CT treatment initiation | | |  | **Model B.** Time to NG/CT-AE reporting | | |
| Variable | Category |  | Treatments/  Person Days | aHR (95% CI) | P-value |  | AEs reported/  Person Days | aHR (95%CI) | P-value |
| Age group in years | 18-24 |  | 92/2482.5 | 1 | - |  | 81/2784.7 | 1 | - |
|  | 25-34 |  | 46/813.1 | 1.08 (0.78-1.49) | 0.656 |  | 29/2298.3 | 0.56 (0.37-0.84) | 0.006 |
|  | 35+ |  | 7/79.1 | 2.20 (1.41-3.45) | 0.001 |  | 6/113.2 | 1.35 (0.61-2.97) | 0.461 |
| School level completed | High school |  | 88/1942.3 | 1 | - |  | 71/3056 | 1 | - |
|  | Primary school |  | 55/1401.8 | 0.51 (0.25-1.05) | 0.067 |  | 43/1975.7 | 0.96 (0.40-2.33) | 0.934 |
|  | No school |  | 2/30.6 | 0.44 (0.2-0.96) | 0.038 |  | 2/164.5 | 0.91 (0.36-2.34) | 0.851 |
| CRS ^c^ | Verulam/Isipingo ^a^ |  | 107/3240.8 | 1 | - |  | 85/4383.3 | 1 | - |
|  | eThekwini ^b^ |  | 38/133.9 | 81.35 (6.64-996.68) | 0.001 |  | 31/812.9 | 2.92 (1.42-6.02) | 0.004 |
| Study visit type at which event occurred ^d^ | Scheduled |  | 100/2607.4 | 1 | - |  | 111/5195.4 | 1 | - |
|  | Unscheduled |  | 45/767.3 | 0.73 (0.44-1.22) | 0.235 |  | 5/0.9 | 1.97 (0.61-6.38) | 0.259 |

Denominators that do not equal the sample sizes are due to missing data.

STI, Sexually Transmitted Infection; CT, *Chlamydia trachomatis*; NG, *Neisseria gonorrhoeae*; TV, *Trichomonas vaginalis*; CRS, Clinical research Site; POC, Point-of-Care;

AE, Adverse Event; aHR, adjusted Hazard Ratio; CI, Confidence Interval.

^a^ Central laboratory-based testing for NG/CT was conducted at the Isipingo and Verulam clinical research sites (CRSs).

^b^ POC testing for NG/CT was conducted at the eThekwini CRS.

^c^ Variable specified as a time-varying-covariate in Models A and B to satisfy proportional hazard assumption.

^d^ Variable specified as a time-varying-covariate in Model B to satisfy proportional hazard assumption.

| **Supplementary Table 6.** Sensitivity analysis with disaggregated CRSs. Cox proportional hazard regression for the impact of POC versus central laboratory-based testing on time to (A) NG/CT treatment initiation and (B) NG/CT-AE reporting among men | | | | | | | | | |
| --- | --- | --- | --- | --- | --- | --- | --- | --- | --- |
|  |  |  | **Model A.** Time to NG/CT treatment initiation | | |  | **Model B.** Time to NG/CT-AE reporting | | |
| Variable | Category |  | Treatments/  Person Days | aHR (95% CI) | P-value |  | AEs reported/  Person Days | aHR (95%CI) | P-value |
| Age group in years | 18-24 |  | 92/2482.5 | 1 | - |  | 81/2784.7 | 1 | - |
|  | 25-34 |  | 46/813.1 | 1.08 (0.78-1.49) | 0.657 |  | 29/2298.3 | 0.56 (0.37-0.85) | 0.006 |
|  | 35+ |  | 7/79.1 | 2.20 (1.40-3.46) | 0.001 |  | 6/113.2 | 1.35 (0.61-2.96) | 0.459 |
| School level completed | High school |  | 88/1942.3 | 1 | - |  | 71/3056 | 1 | - |
|  | Primary school |  | 55/1401.8 | 0.51 (0.25-1.05) | 0.068 |  | 43/1975.7 | 0.96 (0.40-2.31) | 0.928 |
|  | No school |  | 2/30.6 | 0.44 (0.20-0.96) | 0.040 |  | 2/164.5 | 0.90 (0.35-2.34) | 0.837 |
| CRS ^c^ | Isipingo ^a^ |  | 39/1429.5 | 1 | - |  | 29/1564.3 | 1 | - |
|  | Verulam ^a^ |  | 68/1811.3 | 1.00 (0.66-1.53) | 0.992 |  | 56/2819 | 0.97 (0.62-1.51) | 0.888 |
|  | eThekwini ^b^ |  | 38/133.9 | 81.48 (6.22-1067.98) | 0.001 |  | 31/812.9 | 2.87 (1.34-6.11) | 0.006 |
| Study visit type at which event occurred ^d^ | Scheduled |  | 38/133.9 | 1 | - |  | 31/812.9 | 1 | - |
|  | Unscheduled |  | 100/2607.4 | 0.73 (0.44-1.22) | 0.235 |  | 111/5195.4 | 1.96 (0.60-6.4) | 0.265 |

Denominators that do not equal the sample sizes are due to missing data.

STI, Sexually Transmitted Infection; CT, *Chlamydia trachomatis*; NG, *Neisseria gonorrhoeae*; TV, *Trichomonas vaginalis*; CRS, Clinical research Site;

POC, Point-of-Care; AE, Adverse Event; aHR, adjusted Hazard Ratio; CI, Confidence Interval.

^a^ Central laboratory-based testing for NG/CT was conducted at the Isipingo and Verulam clinical research sites (CRSs).

^b^ POC testing for NG/CT was conducted at the eThekwini CRS.

^c^ Variable specified as a time-varying-covariate in Models A and B to satisfy proportional hazard assumption.

^d^ Variable specified as a time-varying-covariate in Model B to satisfy proportional hazard assumption.

**Figure legends**

**Supplementary Figure 1**. Kaplan-Meier cumulative probability graphs displaying the effects of POC versus central laboratory-based testing on NG/CT treatment initiation and NG/CT-AE reporting after sample collection for testing among men.

Supplementary Figure 1A compares the time to NG/CT treatment initiation between CRSs that used either POC (eThekwini) or central laboratory-based testing (Verulam and Isipingo). Supplementary Figure 1B compares the time to NG/CT-AE reporting between CRSs that used either POC (eThekwini) or central laboratory-based testing (Verulam and Isipingo).

Median time in days (95% CI), log-rank test p-value:

**Supplementary Figure 1A** (V-I CRSs= 15.17 (14.17-20.02), eThekwini CRS NG/CT CRS= 0.20 (0.10-0.27), p<0.001). **Supplementary Figure 1B** (V-I CRS= 13.15 (10.21-18.22), eThekwini CRS= 0.20 (0.10-0.27), p<0.001).

V-I CRS, Verulam and Isipingo Clinical Research Sites; NG, *Neisseria gonorrhoeae;* CT, *Chlamydia trachomatis*; AE, Adverse Event; POC, Point-of-Care.
